# Genetic and epigenetic regulation of Catechol-O-methyltransferase in relation to inflammation in chronic fatigue syndrome and Fibromyalgia

**DOI:** 10.1186/s12967-022-03662-7

**Published:** 2022-10-25

**Authors:** Andrea Polli, Jolien Hendrix, Kelly Ickmans, Jelena Bakusic, Manosij Ghosh, Dora Monteyne, Brigitte Velkeniers, Bram Bekaert, Jo Nijs, Lode Godderis

**Affiliations:** 1 Pain in Motion (PiM) international research group, Department of Physiotherapy, Human Physiology and Anatomy, Faculty of Rehabilitation Sciences & Physiotherapy, Vrije Universiteit Brussel, Laarbeeklaan 103, 1090 Jette Brussels, Belgium; 2 Department of Public Health and Primary Care, Centre for Environment & Health, KU Leuven, Kapucijnenvoer 35, 3000, Leuven, Belgium; 3 Flanders Research Foundation–FWO, Brussels, Belgium; 4 Department of Physical Medicine and Physiotherapy, University Hospital Brussels, Brussels, Belgium; 5 Department of Internal Medicine and Endocrinology, University Hospital Brussels, Brussels, Belgium; 6 Department of Forensic Medicine, Laboratory of Forensic Genetics and Molecular Archaeology, University Hospitals Leuven, B-3000 Leuven, Belgium; 7 Department of Imaging & Pathology, KU Leuven, B-3000 Leuven, Belgium; 8 External Service for Prevention and Protection at Work, IDEWE, Heverlee, Belgium; 9 Institute of Neuroscience and Physiology, University of Gothenburg, Gothenburg, Sweden

**Keywords:** Genetics, Epigenetics, DNA methylation, catechol-O-methyltransferase (COMT), Inflammation, Cytokines

## Abstract

**Background:**

Catechol-O-methyltransferase (COMT) has been shown to influence clinical pain, descending modulation, and exercise-induced symptom worsening. COMT regulates nociceptive processing and inflammation, key pathophysiological features of Chronic Fatigue Syndrome and Fibromyalgia (CFS/FM). We aimed to determine the interactions between genetic and epigenetic mechanisms regulating *COMT* and its influence on inflammatory markers and symptoms in patients with CFS/FM. **Methods.** A case-control study with repeated-measures design was used to reduce the chance of false positive and increase the power of our findings. Fifty-four participants (28 patients with CFS/FM and 26 controls) were assessed twice within 4 days. The assessment included clinical questionnaires, neurophysiological assessment (pain thresholds, temporal summation, and conditioned pain modulation), and blood withdrawal in order to assess rs4818, rs4633, and rs4680 *COMT* polymorphisms and perform haplotype estimation, DNA methylation in the *COMT* gene (both MB-COMT and S-COMT promoters), and cytokine expression (TNF-α, IFN-γ, IL-6, and TGF-β). **Results.** COMT haplotypes were associated with DNA methylation in the S-COMT promoter, TGF-β expression, and symptoms. However, this was not specific for one condition. Significant between-group differences were found for increased DNA methylation in the MB-COMT promoter and decreased IFN-γ expression in patients.

**Discussion:**

Our results are consistent with basic and clinical research, providing interesting insights into genetic-epigenetic regulatory mechanisms. MB-COMT DNA methylation might be an independent factor contributing to the pathophysiology of CFS/FM. Further research on DNA methylation in complex conditions such as CFS/FM is warranted. We recommend future research to employ a repeated-measure design to control for biomarkers variability and within-subject changes.

**Supplementary information:**

The online version contains supplementary material available at 10.1186/s12967-022-03662-7.

## Introduction

Understanding the pathophysiology of complex disorders is arguably the first and most important step for developing new therapies aimed at effectively treating complex conditions. Myalgic Encephalomyelitis / Chronic Fatigue Syndrome (CFS) and Fibromyalgia (FM) are two complex conditions characterized by fatigue, widespread pain, and cognitive symptoms affecting 1% and 2–4% of the population, respectively [5, 31]. Diagnostic criteria between the two conditions greatly overlap, and some observations suggest that up to 70% of patients with CFS also comply with the diagnostic criteria of FM [24]. In fact, comorbidity with FM is arguably the best discriminator between CFS patients with high and low symptom burden and disability [24].

The pathophysiology of CFS and FM is poorly understood [18]. The main hypothesis suggests a dysregulation in immune responses and descending nociceptive modulatory mechanisms [4, 23]. One key candidate known to influence both processes is Catechol-O-methyltransferase (COMT) [13]. COMT is an enzyme that degrades catecholamines such as dopamine and noradrenaline, as well as oestrogens [46]. The enzyme is encoded by its homonymous gene, which controls the transcription of two distinct isoforms, a shorter soluble isoform (S-COMT), and a longer membrane-bound isoform (MB-COMT) [46]. Both isoforms are widely distributed in peripheral tissues [28]. The MB-COMT isoform is predominant in the brain, with pronounced activity in the cerebral cortex, hippocampus, and hypothalamus [28]. Three polymorphisms – rs4633, rs4818, and rs4680 – cover particular importance and influence S-COMT enzyme activity [37, 46]. These polymorphisms are inherited together in different haplotypes, which in turn influence COMT activity and clinical presentations [6]. COMT activity is associated with neuroinflammation and hyperalgesia, [6, 20]. as it modulates cytokine expression, particularly TNF-α, IL-1, IL-6, and IFN-γ [8, 13]. Inflammatory cytokines are key in the so-called sickness response, which features signs and symptoms that greatly overlap with the clinical presentation of CFS and FM [49] A recent large study exploring inflammatory markers in patients with CFS reported dysregulation of many cytokines, such as TGF-β, IFN-γ, IL-4, IL-13, and IL-17F [27]. Despite the relatively large amount of research on *COMT* and its role in regulating crucial biological functions, studies exploring the influence of epigenetic mechanisms on downstream inflammation and symptoms in people with CFS and FM are essentially lacking. The present work aims to explore the influence of *COMT* mechanisms on the clinical presentation, descending modulatory mechanisms, and inflammatory markers in patients with CFS/FM. To improve the impact and clinical relevance of the study, we did not limit our investigation to genetic mechanisms but included DNA methylation as well. We expected patients with CFS/FM to show higher expression of inflammatory cytokines, and *COMT* genetic and epigenetic changes to account for cytokine expression.

## Materials and methods

We designed a repeated-measures study with short (four days) between-assessment interval. This design served as an internal validation and allowed us to control for within-subject variability of biological and neurophysiological measures. The study was approved by the Medical Ethical Committee of the University Hospital Brussels (ref. 2016/134), where data collection took place. Data collection was performed between August 2015 and March 2017. Both patients and healthy controls were enrolled in the same period and from the same geographical region. A study exploring different (epi)genetic mechanisms on the same samples of the present research has been recently published elsewhere [38]. The present manuscript is thus not a secondary analyses, but rather explores different biological mechanisms (COMT and cytokines), on different symptoms, and on different neurophysiological aspects (descending modulation).

### Participants

Patients with a clear diagnosis of CFS and FM according to the Centre for Disease Control and Prevention Criteria for CFS, [10] and to the American College of Rheumatology criteria for FM were enrolled [51]. Healthy controls were recruited during the same period among friends or acquaintances of patients or of people involved in the study and matched for sex, age, and body-mass index. Because of the higher prevalence of CFS/FM in women, [39] and because important sex differences exist for pain and immune function, [40] we included only women in the present study. Matching was performed via frequency-matching approach, and checking for frequency of the variables of interest (age and BMI) every 5 patients enrolled in the study. The ratio between patients and controls was 1:1. Participants were excluded if they reported other neurological, psychiatric, cardiopulmonary, or oncological comorbidities. In addition, as physical activity is known to influence both the autonomic nervous system and the immune system, we asked all participants to avoid engaging themselves in physical activity the day before the first assessment or during the study period. As no previous study explored COMT DNA methylation in people with CFS/FM and a repeated measure design, we used one study by Kyoung-Sae et al. [29] to calculate the effect size. Authors explored DNA methylation of COMT in healthy controls and people with clinical depression using our same methodology (PCR-pyrosequencing in the promoter region of MB-COMT). They found a significant between-group mean difference of *1.36% +/- 0.9 (F-test: 28.024*, partial n ^2^: 0.167). Based on that information, G*Power 3.1 calculated an effect size of f = 0.45. We then calculated the sample size needed to detect an effect size of f = 0.45 at the significance level of α = 5% and a power of 80%, using a repeated-measure design with 2 groups and 2 measurements [42]. The total sample size needed was 52 participants. This is also in line with a number of previously published studies measuring variability and clinical relevance of DNA methylation using a candidate gene approach. As summarised by Jones et al [16], most studies show that a mean difference of 1–2% in DNA methylation is sufficient for inducing a significant downstream effect on gene expression [16].

### Procedure

Participants visited the Department of Internal Medicine and Endocrinology of the University Hospital Brussels twice within four days, between 9 and 11 am. Informed consent form was signed before the assessment started. During the first assessment, participants were interviewed. Information regarding general health, comorbidities, and drug intake was noted. Next, they filled in questionnaires assessing physical activity, fatigue, pain, sleep, cognitive and psychological symptoms. Then, a nurse was in charge of blood withdrawal. Samples were processed within one hour from sampling and stored at -80 °C. Afterwards, patients underwent a neurophysiological assessment, as described below. Patients visited our department again after three days, and underwent a second assessment, identical to the first one – questionnaires, neurophysiology, and blood withdrawal. This was done in order to measure reliability of the assessments, as well as to include variability of the biological measures in the statistical models. Assessments prone to assessor-bias were performed blinded to group allocation.

### Clinical and neurophysiological assessment

Clinical characteristics of participants were investigated through the International Physical Activity Questionnaire to measure physical activity, and CFS Symptom List sub-domains to measure the different symptoms associated with CFS: pain, fatigue, sleep disturbances, cognitive problems, and immune aspects (flu-like symptoms). Questionnaires explore clinically relevant aspects for patients with chronic pain and fatigue [33, 48]. They are validated for Dutch-speaking people and show excellent psychometric properties [33, 48]. Neurophysiological assessment included pain sensitivity, endogenous pain facilitation and descending modulation. Neurophysiological mechanisms were assessed by an experimental protocol (lasting approximately 40 min). First, we assessed pain sensitivity by measuring pain threshold to cold, heat and mechanical stimuli. Heat and cold pain thresholds were assessed at three body parts in random order (Neck [on the trapezius superior muscle belly], Hand [on the first inter-digital space of the non-dominant hand], and Leg [on the tibialis anterior muscle belly]) using the TSA-II Neurosensory Analyzer (Medoc, Ltd. Israel). TSA-II is a computerized device designed for measuring sensory thresholds by delivering thermal stimuli to the skin through a 30 × 30 mm thermode. To assess mechanical thresholds, pressure pain thresholds (PPTs) were measured at the hand site only using an analog pressure algometer (Force Dial models FDK 10 Push Pull Force Gate, Wagner Instruments, Greenwich, CT, USA). Three consecutive stimuli were delivered on each body part; participants were asked to stop the stimulation as soon as it was perceived as painful. The thresholds, expressed in °C for the thermal stimulations, and in kg/cm^2^ for mechanical stimulation, are determined as the mean of the last two. They also had to rate the intensity of each stimulus by drawing a vertical line on a visual analogue scale. VAS was represented as a 10-cm horizontal segment going from 0, *No pain*, to 10, *Worst pain imaginable*. These procedures were found to be reliable for measuring hyperalgesia in patients with chronic pain [11]. Endogenous pain facilitation was assessed through a temporal summation protocol, which started 2 min after the final PPT measurement [44, 53]. The measure was taken at the hand site only. Participants were given 10 pulses to the previously determined mean PPT intensity. Pressure was maintained for one second and then released, with 1-second inter-stimulus interval. After the first and tenth pulse, the participant had to rate her pain on the visual analog scale. Temporal summation was calculated as the difference in the pain rate between the 10th and the 1st pulse. Finally, descending modulation was assessed through a conditioned pain modulation paradigm [44, 53]. Participants dipped the dominant hand into mildly-moderate painful (rated between 4/10 and 7/10 on a visual analogic scale) hot water (45 to 46 °C). While the hand was in the water, pain sensitivity was assessed again on the non-dominant hand as described above. Tonic painful stimulation (the hot bath) is the conditioning stimulus and is thought to engage descending inhibitory mechanisms. Descending inhibition is reflected by an increase in pain thresholds on the non-dominant hand while the dominant hand is placed in hot water. Descending modulation is thus measured as the difference in pain threshold (in °C) between before and during hot water immersion. The Polystat Isotemp 4100 C (Fisher Scientific, Aalst, Belgium) was used for accurate controlling of water temperature. The procedure was found to be reliable and valid in healthy people and chronic pain patients, including those with fibromyalgia [3]. Pre-processing of data. Pain threshold measurements for cold and heat stimulation were highly correlated to one another. However, they showed clear ceiling effect. In order to extract the most information from our assessment, we calculated the predicted values imputing the three cold thresholds and the three heat thresholds in two separate regression models. The predicted values were then considered a measure for cold sensitivity and heat sensitivity, respectively (see supplementary material S1 for details).

### Assessment of biological measures

Blood was collected in 4 tubes – 15 ml in 3 EDTA tubes for blood collection and 5 ml in one serum tube – centrifuged (3000 rpm for 10 minutes at 4°C), and stored at -80°C within 1 hour from sampling. DNA was extracted from blood using QIAamp DNA Blood Mini Kit (Qiagen, Hilde, Germany). *DNA methylation and polymorphism analyses.* 200 ng of DNA was incubated with sodium bisulfite using the EZ DNA Methylation-Gold kit (Zymo Research, the Netherlands). Bisulfite-converted DNA sequences of interest were then amplified using polymerase chain reaction (PCR). Genetic and epigenetic analyses were carried out by pyrosequencing – using a Q24 Pyrosequencer device (Qiagen, Hilde, Germany). Pyromark Q24 Analyses software (Qiagen, Hilde, Germany) then measured the polymorphisms and the average methylation in each C-G dinucleotide (CpG) and returned a percentage (from 0–100% methylation) as a result. DNA methylation was measured in MB-COMT promoter region (both before and after the *transcription start site* in Exon I), and S-COMT promoter region (Exon III, overlapping with *translational start site* for both MB-COMT and S-COMT – this region was shown to hold marked regulatory function on S-COMT activity according to the most recent and comprehensive interrogation of the COMT gene) [46]. Three polymorphisms were also assessed via pyrosequencing analyses: rs4633, located in the S-COMT promoter, as well as rs4818 and rs4680, located in Exon IV. More details can be found in the Supplementary Materials (S2). *Haplotype estimation.* Haplotype analysis was performed using Haploview v4.2 [1]. The genetic polymorphisms rs4818, rs4680 and rs4633 passed the default cut-off of the data quality metrics (Hardy-Weinberg equilibrium p-value > 0.001, percentage of non-missing genotypes > 75%, minor allele frequency > 0.001) and were thus all included for haplotype analysis. Markers of linkage disequilibrium (D’, r² and LOD) were calculated automatically by Haploview v4.2, which derives these markers from maximum-likelihood values of the four gamete frequencies estimated via a two marker Expectation – Maximization (EM) algorithm [1]. The genetic polymorphisms were manually grouped into one block after which haplotype association analysis was performed. Differences in genotype or haplotype frequency distribution were tested using the Chi Square test. See Supplementary Materials (S2) for details. *Inflammatory cytokines.* Enzyme-Linked Immunosorbent Assay (ELISA) human assay kit (Thermofisher, Invitrogen Inc., USA) was used to measure TNF-α, IL-6, IFN-γ, and TGF-β in serum. Invitrogen products ensure high reliability (average intra-assay coefficient of variation: 4.5%; inter-assay variability: 5.7%; no detectable cross-reactivity). Details on the validation procedure can be found in the Supplementary Materials (S2). Of note, a number of samples could not be detected with ELISA, possibly due to degradation of the samples. IL-6 was detected in 99, INF-γ in 94, and TGF-β in 102 out of 108 samples. TNF-α was not detectable.

### Statistical analyses

Explorative analyses were performed to assess distribution, skewness, variance of continuous variables, and to identify possible covariates. Between-assessment stability of neurophysiological and biological measures were assessed using the interclass correlation coefficient (ICC). Then, repeated-measure linear mixed models (RM-LMM) were employed. RM-LMM allowed us to increase power and precision of our observations, as they take within-subject measure variability into account. Separate models were built to assess between-group differences and the effect of the haplotypes on symptoms, hyperalgesia, descending modulation, inflammation, and mean DNA methylation (for each primer separately), as well to assess the association between DNA methylation and symptoms, inflammation, hyperalgesia, and descending modulation. As commonly recommended, [47] we started with a *basic model* including Subjects as a random term, Time as repeated measure, Group as categorical factor, age, BMI, and symptoms as covariates. Additional covariates (haplotype, DNA methylation, cytokines) were then progressively added. Each time, model’s *goodness-of-fit* was assessed by the *− 2 Restricted Log Likelihood.* Only models with the best fit were considered in further analyses. Bonferroni correction was applied to reduce the risk of false positives for multiple comparisons. P-values of Fixed-Effect tests were also adjusted, using the formula: *Corrected P-value = 0.05/n° of predictors* [47]. P-values were thus set at 0.008. All data were analyzed with SPSS 27 (Chicago, IL, USA).

## Results

As previously described, [38] fifty-four participants completed the two assessments – 28 women with CFS/FM and 26 healthy controls. Mean age was 49.9 (SE = 1.4), and mean body-mass index (BMI) was 24.4 (SE = 0.6). No evidence of between-group differences for measures of temporal summation or CPM were found. As expected, patients reported significantly more symptoms than controls (F = 180.602, p < .001; β = 77.922, 95% C.I. 66.407 to 89.436) (Table 1).

**Table 1:** **Description of healthy controls (HC) and patients with chronic fatigue syndrome and fibromyalgia (CFS/FM) including clinical data, measures from conditioned pain modulation, cytokine expression, DNA methylation, and prevalence of polymorphisms**. Data is reported as the mean estimate and standard error (SE) of both assessments (at Day 1 and Day 4) from the repeated measure mixed linear models and include subjects as random factor, time as repeated effect, group and haplotype as fixed effects, and age, body-mass index and symptoms at the CSL questionnaire as covariates, as described in the statistical analyses. CSL: chronic fatigue syndrome symptom list. CPM: conditioned pain modulation (^#^ numbers should be interpreted as the higher the number the better the descending modulation); IL-6: interleukin-6; IFN-γ: interferon-γ; TGF-β: transformative growth factor-β; COMT: Catechol-O-methyltransferase. C.I.: confidence interval. *between-group significant differences at p < .05. χ: Chi-squared Test’s value

	HC n = 26	CFS/FM n = 28	Mean difference (95% C.I.)	p
**Clinical**				
CSL Total	23.51 (5.04)	99.80 (5.69)	76.29 (61.88; 90.69)	< 0.001*
**CPM (°C)** ^**#**^				
To cold stimuli	1.89 (0.37)	2.16 (0.43)	0.271 (-0.787; 1.33)	0.610
To heat stimuli	0.92 (0.28)	0.98 (0.31)	0.059 (-0.71; 0.82)	0.878
**Cytokines**				
IL-6 (pg/mL)	0.71 (0.26)	1.30 (0.22)	0.591 (-0.135; 1.32)	0.109
IFN-γ (pg/mL)	12.29 (1.22)	6.62 (1.50)	-5.66 (-9.70; -1.62)	0.007*
TGF-β (ng/mL)	12.18 (1.03)	13.07 (1.12)	0.89 (-2.44; 4.22)	0.597
**DNA Methylation (%)**				
MB-COMTa	2.39 (0.183)	2.78 (0.21)	0.39 (-0.14; 0.94)	0.465
MB-COMTb	1.55 (0.12)	1.54 (0.13)	0.07 (-0.53; 0.52)	0.978
MB-COMTc	1.70 (0.29)	3.12 (0.31)	1.42 (0.52; 2.32)	0.003*
Mean MB-COMT	1.94 (0.22)	2.74 (0.23)	0.80 (0.14; 1.45)	0.018*
S-COMTa	61.63 (1.22)	62.47 (1.37)	0.81 (-3.23; 4.85)	0.691
S-COMTb	93.39 (0.36)	93.90 (0.39)	0.50 (-0.53; 1.53)	0.334
S-COMTc	91.74 (0.34)	92.18 (0.38)	0.44 (-0.68; 1.56.71)	0.436
Mean S-COMT	81.13 (0.50)	81.71 (0.56)	0.58 (-1.07; 2.23)	0.487
**Polymorphism (n)**				
rs4633 (TT/CT/CC)	4/15/7	4/21/3	2.529^χ^	0.287
rs4818 (CC/GC/GG)	5/15/6	7/16/5	.072^χ^	0.965
rs4680 (AA/GA/GG)	4/15/7	5/20/3	2.930^χ^	0.403

### Relevance of COMT haplotypes

**Fig. 1:**
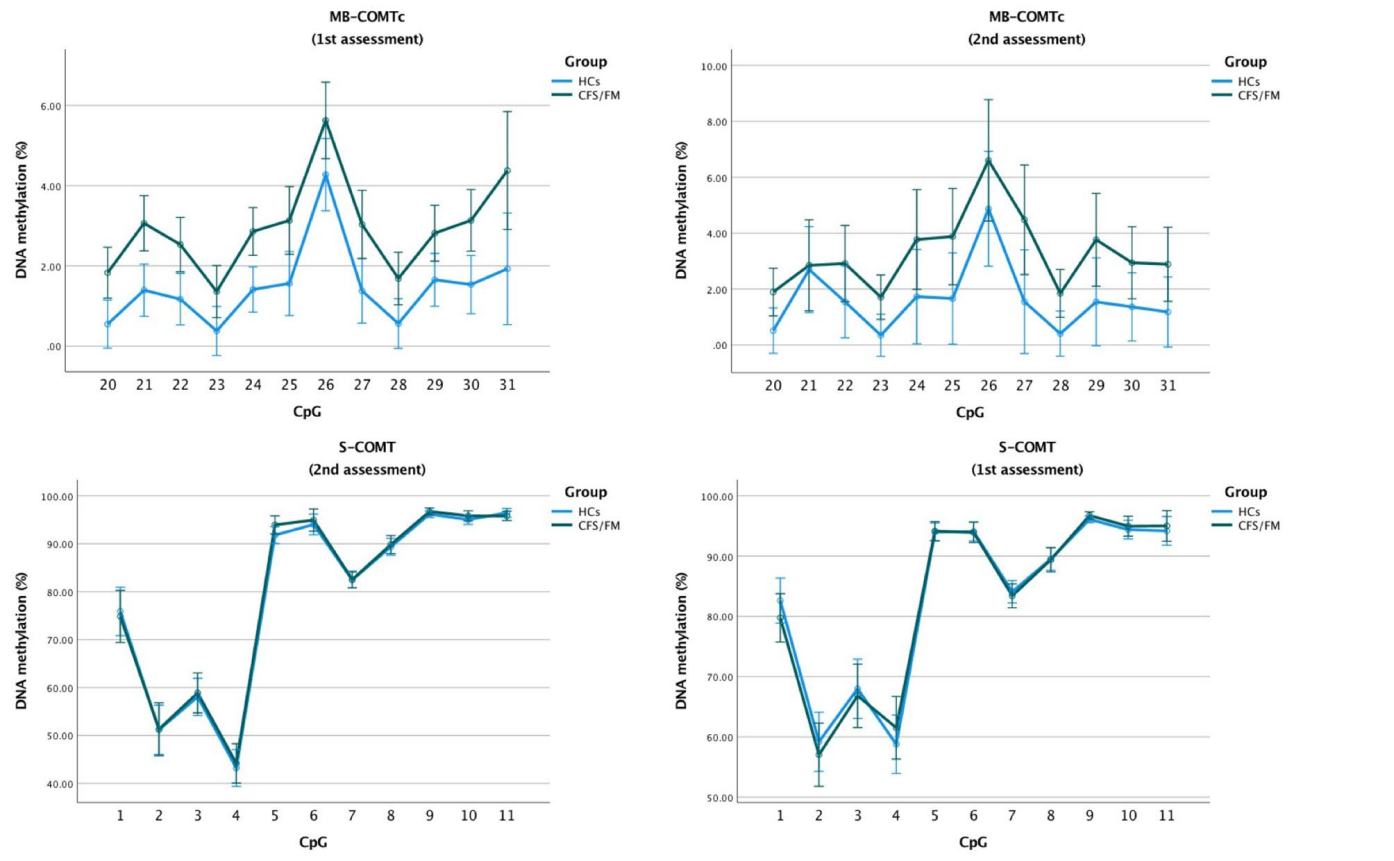
**DNA methylation for each CpG at promoter MB-COMT(c) and S-COMT in healthy controls (HC) and patients with Chronic Fatigue Syndrome and Fibromyalgia (CFS/FM), at both assessments.** Statistically significant difference for mean methylation were found only at the MB-COMT promoter. Error bars represent 95% confidence interval.

The three polymorphisms show similar prevalence in both groups (see Table 1), this in line with the previously observed prevalence in Caucasian population [36]. Haplotype estimation confirmed previous observations that the polymorphisms are transmitted in haplotype [41]. (Table 2). We identified 4 haplotypes per allele in our sample, which we then combined and estimated COMT enzyme activity, as reported in previous research [30]. Following previously published research, [30]. the 4 haplotypes corresponded to “High COMT activity”, “High-intermediate COMT activity” “Intermediate COMT activity” and “Intermediate-low COMT activity”. These 4 categories were then used for further analyses. Haplotypes showed a clear association with DNA methylation levels at the S-COMT promoter, particularly in the first part of the region (F = 11.539, p < .001; high vs. Intermediate-low activity haplotype: β= − 13.002%, 95% C.I. − 21.310 to − 4.694) (Table 3; Fig. 1). This suggests that low activity haplotypes might influence DNA methylation, reducing mean DNA methylation. Higher DNA methylation in turn contributes to lower COMT activity. COMT haplotype was significantly associated with the expression of one cytokine, TGF-β (F = 5.163; p = .003; high vs. Intermediate-low activity haplotype: β= − 7.712 ng/mL, 95% C.I. − 14.497 to –0.927). Finally, COMT haplotype also had an effect on symptoms (F = 3.795, p = .014; post-hoc analyses high vs. intermediate-low COMT activity: β= − 44.771, 95% C.I. − 87.889 to − 1.652) (Table 2), and cold thresholds (F = 4.468, p = .003; high vs. Intermediate-low activity haplotype: β= − 9.26, 95% C.I. − 16.197 to − 2.330).

**Table 2:** **Effect of COMT haplotypes on symptoms at the Chronic Fatigue Syndrome symptom list (CSL), pain thresholds, and conditioned pain modulation in both healthy controls (HC) and patients with Chronic Fatigue Syndrome and Fibromyalgia (CFS/FM).** Data is expressed as estimated adjusted mean (standard error). Mean difference and p-values refer to the difference between the first and the last category. CPT: cold pain thresholds; HPT: heat pain thresholds; C-CPM: conditioned pain modulation to cold stimuli; H-CPM: conditioned pain modulation to heat stimuli

	Healthy controls				Patients with CFS/FM					
	**COMT** **High** **activity**	**COMT** **High-intermediate** **activity**	**COMT** **Intermediate** **activity**	**COMT intermediate-low** **activity**	**COMT** **High** **activity**	**COMT** **High-intermediate** **activity**	**COMT** **Intermediate** **activity**	**COMT intermediate-low** **activity**	**Mean diff.** **(95% C.I.)**	**p**
CSL Total	18.78 (8.85)	22.54 (5.55)	28.86 (8.59)	63.55 (13.02)	91.83 (10.73)	95.59 (5.08)	101.91 (8.93)	136.60 (11.95)	-44.771 (-87.89; -1.65)	0.014*
CPT (°C)	2.10 (1.51)	5.24 (1.15)	4.49 (1.42)	11.36 (2.15)	5.47 (1.61)	8.61 (0.95)	7.83 (1.48)	14.74 (2.38)	-9.263 (-16.197; -2.33)	0.006*
HPT (°C)	47.55 (0.95)	47.19 (0.72)	47.73 (0.89)	46.45 (1.35)	45.80 (1.01)	45.44 (0.60)	45.97 (94)	44.69 (1.50)	1.108 (-3.26; 5.47)	0.825
C-CPM (°C)	2.19 (0.56)	2.43 (0.41)	2.05 (0.50)	4.99 (0.77)	1.97 (0.57)	2.22 (0.34)	1.84 (0.52)	4.78 (3.02)	-2.804 (-5.39; − 0.21)	0.011*
H-CPM (°C)	1.23 (0.40)	1.14 (0.30)	1.17 (0.37)	2.10 (0.56)	0.89 (0.42)	0.81 (0.25)	0.84 (0.37)	1.76 (0.62)	− 0.872 (-2.68; 0.93)	0.418

**Table 3:** **Effect of COMT haplotype on DNA methylation in the whole group.** Data is reported as mean (standard error) of both assessments. F-test and p-values refer to the univariate test of the Linear Mixed Model

	Haplotype					
**DNA methylation (%)**	**High COMT activity**	**High-intermediate COMT activity**	**Intermediate COMT activity**	**Intermediate-low COMT activity**	**F**	**p**
MB-COMTa	2.79 (0.34)	2.88 (0.18)	2.51 (0.29)	2.52 (0.56)	0.557	0.646
MB-COMTb	1.43 (0.21)	1.80 (0.12)	1.45 (0.19)	1.51 (0.33)	1.73	0.170
MB-COMTc	2.15 (0.36)	2.36 (0.19)	2.30 (0.37)	2.84 (0.56)	0.355	0.785
MB-COMT mean	2.25 (0.27)	2.48 (0.15)	2.21 (0.26)	2.41 (0.41)	0.495	0.687
S-COMTa	54.42 (1.57)	60.42 (0.74)	66.00 (1.41)	67.43 (2.52)	11.539	< 0.001*
S-COMTb	91.95 (0.64)	93.09 (0.31)	94.80 (0.57)	95.97 (1.01)	5.879	0.001*
S-COMTc	91.71 (0.44)	92.52 (0.22)	92.15 (0.39)	91.47 (0.70)	1.573	0.202
S-COMT mean	78.21 (0.64)	80.91 (0.30)	83.02 (0.58)	83.54 (1.03)	11.580	< 0.001*

### DNA methylation, cytokines, and symptoms

**Fig. 2:**
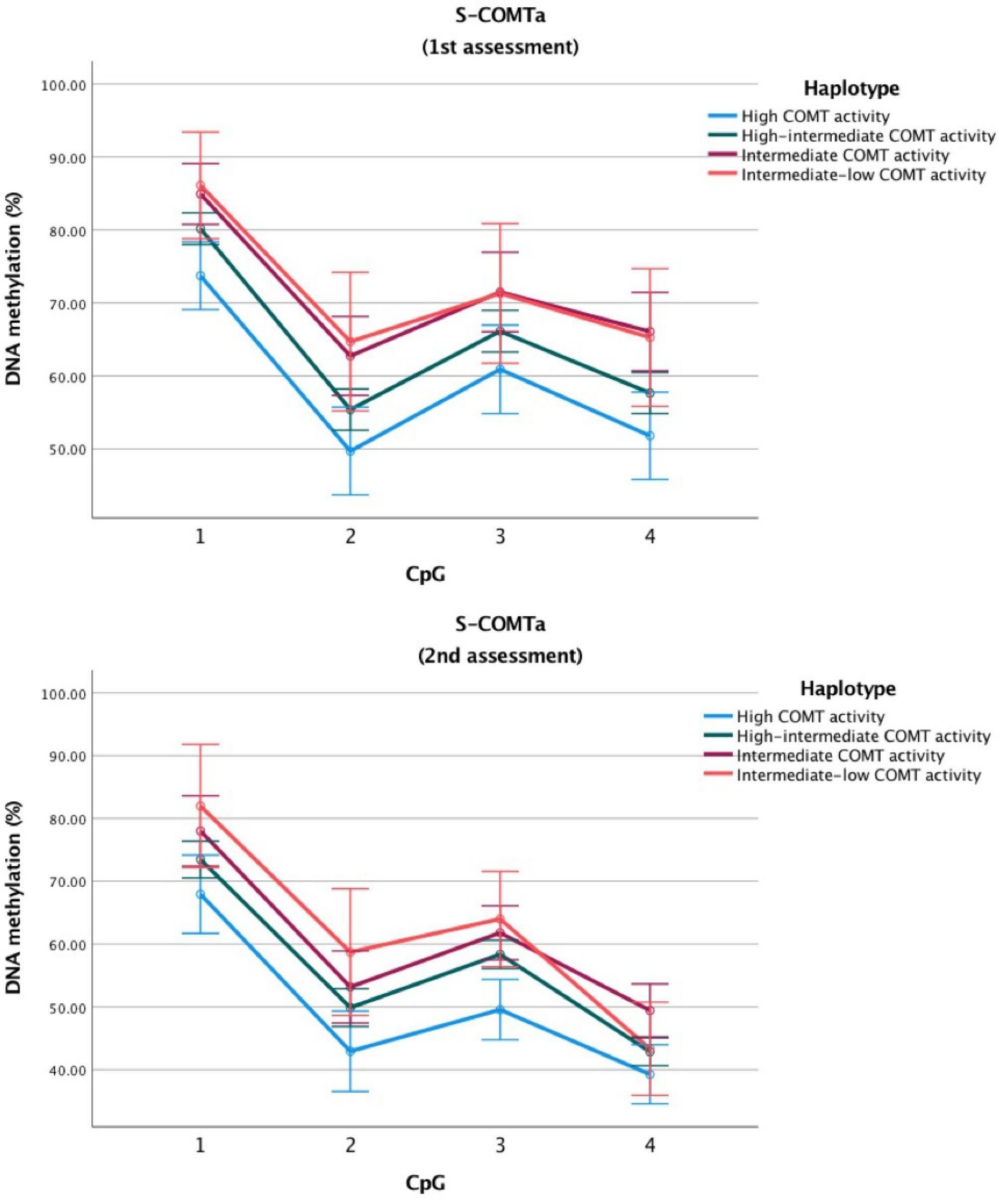
**DNA methylation across the estimated COMT haplotypes for each CpG at S-COMT(a) in the whole group at both assessments.** Error bars represent 95% confidence interval. See Table 3 for additional details.

With respect to DNA methylation, significant between-group differences were found only in the MB-COMT promoter region, which showed higher methylation in patients, as compared to healthy controls. It is particularly the third region of the promoter, MB-COMTc, that drives the effect (F = 9.830, p = .003; β = 1.418%, 95% C.I. 1.112 to 2.81, Table 1; Fig. 2). Patients showed about double the DNA methylation seen in healthy controls. IFN-γ was found to be significantly lower in patients with CFS/FM (F = 9.840, p = .003; β= − 6.562 pg/ml, 95% C.I. − 10.757 to − 2.367). No significant associations were found between DNA methylation and cytokines, nor between DNA methylation and symptoms. Age was significantly associated with a reduction of DNA methylation in MB-COMTb (F = 8.470, p < .005; β= –0.023%, 95% C.I. –0.039 to –0.007). Age (F = 17.238, p < .001; β= –0.323 pg/ml, 95% C.I. –0.479 to –0.167) and IL-6 (F = 11.494, p = .001; β = 2.151 pg/ml, 95% C.I. 0.885 to 3.417) showed an effect on IFN-γ expression. Neither group nor haplotype affected IL-6 concentration. Significant effects on IL-6 concentration were found for INF-γ (F = 14.335, p < .001; β = 0.067 pg/ml, 95% C.I. 0.031 to 0.102).

## Discussion

To the best of our knowledge, this is the first investigation comprehensively assessing the interactions among different aspects: COMT haplotypes, DNA methylation, inflammation, and symptoms in any clinical population. We employed a repeated-measure design that served as an internal control, improved statistical power, and overcame most limitations typical of cross-sectional studies. Our statistical models were conservative and p-values were adjusted (α = 0.008) to reduce the occurrence false positives. We found a significant association between COMT haplotypes and DNA methylation. Haplotype associated with higher COMT enzyme activity showed lower DNA methylation in S-COMT, but not in the MB-COMT promoter region. This was found in both the CFS/FM and the healthy control group. S-COMT has been shown to be the region that influences enzymatic activity the most [37, 46]. Associations between genetic variability and DNA methylation have been observed in many genes [12]. Both gene polymorphisms and DNA methylation state, as well as the interaction between the two, are able to influence gene expression [12]. Lower COMT activity has in fact been associated with increased catecholamine levels in the brain, which in turn have been associated with pain, hyperalgesia, and increased susceptibility to infections [6, 19, 20], all established features of CFS/FM.[25, 32] Our results are in line with these findings, as we showed the low-activity haplotype to be associated with symptoms in both patients with CFS/FM and healthy controls. Lower-activity haplotype was also associated with TGF-β expression. TGF-β is a cytokine with diverse functions, one of which being a pro-inflammatory action and the activation of T-helper 17 cells, a group of pro-inflammatory T-cells. TGF-β was also found elevated in CFS in a recently published study [27].

Patients with CFS/FM showed higher DNA methylation at the MB-COMT promoter. Transcriptional regulation of MB-COMT is less clear, though recent evidence suggests that DNA methylation within MB-COMT promoter region reduces enzyme transcription [52]. As previously showed by others, [37, 46] we found that MB-COMT methylation is independent from the studied polymorphisms and haplotype. Thus, higher methylation levels found in patients with CFS/FM would result in lower enzymatic activity and worse symptoms. MB-COMT activity is of particular importance in the brain, where S-COMT is less expressed [28]. However, no studies investigated MB-COMT methylation relevance in disorders characterized by persistent pain or fatigue. COMT is able to influence both neuroinflammation and immune system functioning, as well as the availability of catecholamines and other neurotransmitters, such us endogenous opioids, and hormones [55]. Our findings are in line with the research suggesting that neuro-immune alterations contribute to the pathophysiology of CFS and FM [17, 19, 26, 43].

Another significant result was a lower expression of IFN-γ in patients. IFN-γ has long been known as an inflammatory cytokine, e.g. by inducing the production of other cytokines such as IL-6 and TNF-α [34]. However, other observations highlight the role of IFN-γ as master regulator of immunity [54]. Low levels of IFN-γ (below 50 pg/ml) exert a potent anti-inflammatory effect [9]. Our results seems to confirm the anti-inflammatory role of IFN-γ, as already showed by previous research in CFS [2]. However, given some conflicting results present in literature, [34] further research is needed to confirm our findings, better if employing, when possible, assays for full cytokine profiling.

We acknowledge that the study has some limitations, mostly related to the relatively small sample size. It is possible that some associations between DNA methylation and symptoms were not detected due to lack of appropriate power. For instance, we found no significant association between COMT DNA methylation and CFS/FM symptoms or IFN-γ and IL-6 expression. Another limitation concerns the generalizability of our results. As explained, given prevalence of CFS and FM is much higher in women (70–80%)[39] and because important sex differences exist for pain and immune function, [40], we included only women in the present study. This might mean that our finding might differ in men with CFS/FM, and we should be careful when trying to generalize current results to a male population with CFS/FM. Finally, including more direct measures of COMT activity (by directly measuring COMT activity in viable cells or measuring catecholamine expression and their metabolites) would give extra-, more accurate information, on the effect of DNA methylation on catecholamine expression. It is catecholamines and their interaction with adrenergic receptors that ultimately induce pro-inflammatory cytokines release [13]. High levels of catecholamine expression can also have broad effects on other biological mechanisms, including oxidative stress and mitochondrial function [21]. Mitochondrial dysfunctions and metabolic alterations have been repeatedly observed in people with CFS/FM.[15,45,50] Research is not yet conclusive, and the cause of such metabolic alterations is unclear. Our results might suggest that mechanisms related to the autonomic nervous system might play a role. Further research should focus on the relation between catecholamine expression and mitochondrial function to provide potentially crucial pathophysiological mechanisms [14]. This might be especially interesting in light of recent advancements in the field of biomaterials and nano-antioxidants, which appear to interact with inflammatory genes [,^35^].

Finally, we feel the urge to highlight that translational research is extremely complex in nature. Dealing with often remarkably variable biological markers is challenging. This is especially true when researchers attempt to better understand complex and heterogeneous conditions such as CFS/FM, where multiple complex mechanisms are likely at play and influence each other. We recommend future research to employ a repeated-measure design to control for biomarkers variability and within-subject changes. In addition, a better characterization of the subjects enrolled in the research is warranted. Cognitive, psychological, and social aspects can all interact with biological, behavioural, and psychophysical assessments and needs to be considered when designing a clinical study [7].

## Conclusion

Overall, our results confirm previously published research on the role of COMT haplotypes and their interaction with S-COMT promoter, inflammatory markers, and symptoms. However, prevalence of COMT polymorphisms and haplotypes is similar in patients and controls, thus suggesting that it is not a specific aspect of CFS/FM. On the contrary, DNA methylation at the MB-COMT promoter, as well as the anti-inflammatory actions exerted by IFN-γ, might hold clinical relevance in patients with CFS/FM. Research on DNA methylation and biomarkers in complex conditions such as CFS/FM is warranted and holds promise for detecting new targetable mechanisms.

## Electronic supplementary material

Below is the link to the electronic supplementary material.

Supplementary Material 1

Supplementary Material 2

## Data Availability

The complete dataset is deposited in the repository of the Vrije Universiteit Brussel. (VUB) and can be made available via a request form to the VUB and the Principal. Investigator.
