# Supplementary material for: Genetic and epigenetic regulation of Catechol-O-methyltransferase in relation to inflammation in chronic fatigue syndrome and Fibromyalgia
Source: J Transl Med. 2022 Oct 25;20:487. doi: 10.1186/s12967-022-03662-7 (PMC9598022; doi:10.1186/s12967-022-03662-7)
Supplement: Supplementary file 1 — Supplementary Material 1 [file 12967_2022_3662_MOESM1_ESM.pdf]

**SUPPLEMENTARY MATERIAL (S1)**
**PRE-PROCESSING OF PAIN THRESHOLD MEASUREMENTS**

Pain thresholds to cold and heat showed clear ceiling effect. Many healthy controls and several patients reached the stimulation ceiling of the TSA-II device (50°C for heat stimulation, 0°C for cold stimulation). Clear and significant between-group differences could still be detected at both assessments. We report below the table S1-1, as also reported elsewhere.[3]

**Table S1-1. Between-group differences on pain thresholds.** CFS: Chronic Fatigue Syndrome; HC: healthy control; CT: Cold Threshold; HT: Heat Threshold. *: statistically significant at the univariate F-test for the effect of Group using Repeated-measures mixed models. The effect of time, age and BMI was non-significant in the models. Data are expressed as mean and standard deviation.

| | **1st Assessment** | | **2nd Assessment** | | **Group diff.** | |
|---|---|---|---|---|---|---|
| | CFS (n=28) | HCs (n=26) | CFS (n=28) | HCs (n=26) | F-test | Sig. |
| Hand CT | 9.84 (8.05) | 3.80 (5.08) | 8.99 (7.37) | 3.29 (4.93) | 18.975 | .001* |
| Hand HT | 45.14 (4.63) | 47.08 (3.70) | 45.65 (4.25) | 48.10 (2.46) | 9.695 | .002* |
| Leg CT | 7.42 (10.19) | 3.30 (7.05) | 7.86 (10.69) | 2.78 (6.01) | 7.713 | .007* |
| Leg HT | 47.64 (2.41) | 48.47 (2.51) | 48.06 (1.95) | 49.28 (0.91) | 6.734 | .011* |
| Neck CT | 10.47 (11.19) | 4.35 (8.46) | 10.84 (11.17) | 2.94 (6.23) | 11.318 | .001* |
| Neck HT | 46.75 (3.92) | 48.46 (2.86) | 47.13 (3.78) | 48.96 (1.81) | 9.845 | .002* |

However, **conditioned pain modulation (CPM),** relies on detecting the effect of a painful conditioning stimulus (hot water immersion in our case) on a test stimulus (hot and cold pain threshold). Pain thresholds are supposed to increase while the contralateral hand is receiving tonic painful stimuli (hot water bath). Therefore, ceiling effect would mask the effect of the conditioning stimulus, as the highest possible value is reached before the CPM paradigm. In fact, CPM effect was found statistically significant only in the patient group ($p<.05$ at within-group paired test, when comparing thresholds before and after CPM). Data inspection revealed that this was clearly due to the ceiling effect seen in the healthy control group.

As correlation analyses showed among pain thresholds were moderately correlated in both the healthy control and the CFS/FM group (all $r>.400$), we used predictive values, rather than actual values, for further analyses. Briefly, we created two separated regression models (one for cold thresholds and one for heat thresholds) with pain threshold at the hand as a dependent variable, and pain threshold at the leg and neck as independent variable. The hand site was chosen as a dependent variable because it was the most reliable of the three measures, according to the interclass correlation coefficient (hand cold threshold: $\alpha=.935$, $p<.001$; hand heat threshold: $\alpha=.915$, $p<.001$). The models calculated a regression coefficient and returned a predicted value which was based on the pain threshold at the hand site, but considered the other two thresholds as well. In this way, we were able to create two new variables that we considered a measure for cold sensitivity and heat sensitivity, respectively. Predicted values indeed showed less ceiling effect and were

less skewed towards the limit values (0°C for cold thresholds and 50°C for heat threshold). See table S1-2 below.

**Table S1-2. Between-group differences on pain thresholds predicted values.** CFS: Chronic Fatigue Syndrome; HC: healthy control; PCT: Predicted Cold Threshold; PHT: Predicted Heat Threshold. CPM: conditioned pain modulation; #: statistically significant at the paired t-test comparing values before and after CPM; *: statistically significant at the univariate F-test for the effect of Group using Repeated-measures mixed models. The effect of time, age, and BMI was non-significant in the models. Data are expressed as mean and standard deviation.

| | **1st Assessment** | | **2nd Assessment** | | **Group diff.** | |
|---|---|---|---|---|---|---|
| | CFS (n=28) | HCs (n=26) | CFS (n=28) | HCs (n=26) | F-test | Sig. |
| **PCT** | 8.63 (6.37) | 5.11 (4.85) | 8.12 (5.62) | 4.23 (3.14) | 14.178 | .001* |
| **PCT-CPM** | 5.57 (5.05) | 3.04 (3.24) | 5.54 (4.94) | 2.67 (2.90) | | |
| **Sig.** | .001# | .001# | .001# | .001# | | |
| **PHT** | 45.23 (3.72) | 46.98 (3.10) | 45.83 (3.52) | 47.90 (1.36) | 11.794 | .001* |
| **PHT-CPT** | 46.66 (2.94) | 48.17 (1.84) | 46.92 (2.63) | 48.31 (0.72) | | |
| **Sig.** | .003# | .001# | .006# | .033# | | |

Between-group difference were still significant at both timepoints on both independent samples t-tests, and in repeated-measure mixed linear model (Table S1-2). Standard deviation was reduced and did not overlapped with the device limit values anymore. In addition, within-group paired t-tests showed that CPM had a significant effect on both patients with CFS/FM and controls (see Table S1-2).

We believe that this statistical approach to pain thresholds is able to retrieve the most useful information without distorting the original data, and should be considered when ceiling effect is negatively influencing pain thresholds assessment.

## Bibliography for Supplementary Material S1


[1] Cathcart S, Winefield AH, Rolan P, Lushington K. Reliability of temporal summation and diffuse noxious inhibitory control. Pain Res Manag 2009;14:433–438.

[2] Graven-Nielsen T, Arendt-Nielsen L. Peripheral and central sensitization in musculoskeletal pain disorders: an experimental approach. Curr Rheumatol Rep 2002;4:313–21. doi:10.1007/s11926-002-0040-y.

[3] Polli A, Ghosh M, Bakusic J, Ickmans K, Monteyne D, Velkeniers B, Bekaert B, Godderis L, Nijs J. DNA methylation and BDNF expression account for symptoms and widespread hyperalgesia in patients with Chronic Fatigue Syndrome and Fibromyalgia. Arthritis Rheumatol 2020.

[4] Staud R, Robinson ME, Price DD. Temporal Summation of Second Pain and Its Maintenance Are Useful for Characterizing Widespread Central Sensitization of Fibromyalgia Patients. J Pain 2007;8:893–901.

[5] Yarnitsky D, Arendt-Nielsen L, Bouhassira D, Edwards RR, Fillingim RB, Granot M, Hansson P, Lautenbacher S, Marchand S, Wilder-Smith O. Recommendations on terminology and practice of psychophysical DNIC testing. Eur J Pain 2010;14:339. doi:10.1016/j.ejpain.2010.02.004.

## SUPPLEMENTARY MATERIAL (S2)
## VALIDATION PROCEDURES FOR BIOLOGICAL MEASUREMENTS

*PCR and Sequencing Primer design*

For COMT DNA methylation analyses, we used PyroMark Assay Design Software 2.0 to design forward, reverse, and sequencing primers in order to assess DNA methylation in three regions of interest – MB-COMT, S-COMT, and Exon IV (where two out of three polymorphisms are located – rs4818 and rs4680). Regions of interest were selected based on previous literature, which showed these regions to influence gene expression.[2,3] Using a gradient PCR Device (Veriti 96-Well Thermal Cycler, Applied Biosystems, ThermoFisher Scientific, Belgium), we undertook the validation process to detect which annealing temperature worked best. PCR cycling protocol was set as follows:

- One activation step: 95°C for 15 minutes,
- 45 cycles including 3 steps
    - 30 seconds at 94°C for denaturation,
    - 30 seconds at the annealing temperature detected during the validation procedure (see table S2-1 below for details)
    - 30 seconds extension step at 72°C
- One final extension step of 10 minutes at 72°C

**Table S2-1. Primers for DNA methylation analyses of the COMT gene.** MB-COMTa, MB-COMTc, and S-COMTc have the forward primer biotinylated, while MB-COMTb, S-COMTa, S-COMTb, and the primers designed for Exon IV have the reverse primer with biotin. Temperatures refer to the annealing temperatures used for PCR amplification.

| Name | Fwd-PCR-primer | Rev-PCR-primer | Sequencing primer | CpGs | Temp |
|---|---|---|---|---|---|
| MB-COMTa | GTTGAGGGG ATTAGGAGGG | CCCCAATTT CCCCACCTA | CCCATCCT ACCTACT | 13 | 58°C |
| MB-COMTb | GGGTAGTTTTAGT TGTTTTAGAAGTTT | ACCCCATCCTA CCTACTAC | CACCCAAAACCCC CTCCTAATC | 6 | 58°C |
| MB-COMTc | TGGGGTAGTTAG GGTTGT | ATCTAACCAACC CTCTCACCTCTC | CAACCCTCTCACCTCTCC | 11 | 58°C |
| S-COMTa | GGATGGGTTG TAGGATGAAT | GGATGGGTTG TAGGATGAAT | AGTAATATAGTTG TTAATAGTAGA | 4 | 54°C |
| S-COMTb | GTGGATGGG TTGTAGGATGA | AAACCCCCCCTCTA CTATTAACAACTAT | GGGTTGTAGG ATGAAT | 2 | 54°C |
| S-COMTc | TGTTTATGGGTGA TATTAAGGAGTAG | TCATAACCCACT CCTTCTACT | AAATATCAAT AACCTCCAAC | 6 | 54°C |
| Exon IV | TGGGGGTTTATT GTGGTTAT | AACTATAAAACCCT CACTAAACTACTA | ACACACCTTATCCTTC | 6 | 56°C |

*PCR Primer Validation*

Figure S2-1 below report details on the primer validation procedure. We reported only two examples, in order to clarify the procedure. The same procedure applies to all primers.

**Figure S2-1. Examples of gel electrophoresis (above) and electropherogram for selected temperatures (below).** Figures represent PCR product amplification for MB-COMT (columns B) and S-COMT (columns C). Numbers 1 to 6 refer to different temperatures, respectively: 50°C, 52°C, 54°C, 56°C, 58°C, 60°C. Based on the graphs below, 58°C (B5) was chosen for MB-COMT and 54°C (C3) was chosen for S-COMT.

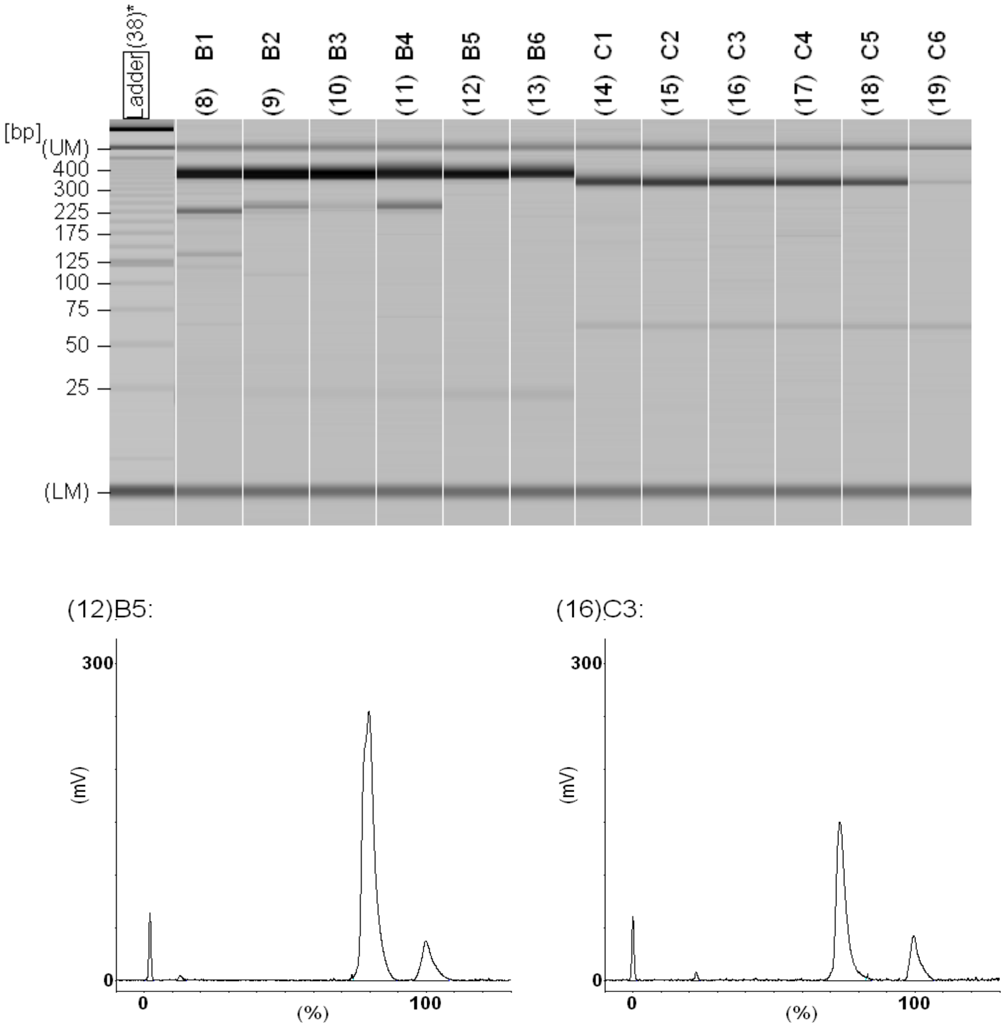


*Randomisation and positive controls for pyrosequencing analyses*

Pyrosequencing was performed on 24-well plates using a Q24 Pyrosequencer device (Qiagen, Hilde, Germany). Sample randomisation was performed in order to reduce the bias that might have accumulated during the lab procedures, and to ensure that each plate would include both patients and controls in random order. DNA positive (highly methylated) controls used to validate the pyrosequencing. We included two wells per analysis, in different plates (see Table S2-2 below). One of the two controls in the MB-COMTa plate did not work and variability could not be calculated.

**Table S2-2**. **Mean methylation for each positive control in the two wells, and the inter-sample variability.** Positive controls were expected to be highly methylated (>80%) and with low variability (up to 5%). Only MB-COMTa and Exon IV did not reach 80% methylation. However, variation was small so this will unlikely impact on the analyses. Plus, even in the case of higher variation, all samples are randomised, and the repeated measure design allowed us to test each subject twice.

| DNA region | Positive control 1 | Positive control 2 | Variability |
|---|---|---|---|
| MB-COMTa | 71.85% | / | / |
| MB-COMTb | 86.49% | 86.99% | 0.50% |
| MB-COMTc | 86.18% | 82.33% | 3.85% |
| S-COMTa | 94.00% | 90.18% | 3.82% |
| S-COMTb | 100.00% | 98.61% | 1.39% |
| S-COMTc | 84.52% | 84.77% | 0.22% |
| Exon IV | 72.56% | 74.37% | 1.81% |

*Frequency and haplotypes for the genetic polymorphisms rs4818, rs4633, and rs4680*

There was a strong linkage disequilibrium between the genetic polymorphisms rs4818, rs4680 and rs4633 (Table S2-3), indicating that they are co-inherited most of the time. The frequencies of the observed genotypes for rs4633 (CC/CT/TT), rs4818 (CC/GC/GG), and rs4680 (AA/GA/GG) were similar in healthy controls and patients with CFS/FM (Table S2-4). Haplotypes were not associated with CFS/FM as was shown by the similarities in individual haplotype frequencies and overall distribution of the haplotype frequencies (analysis via SPSS) between healthy controls and patients with CFS/FM (Table S2-5)

**Table S2-3. Linkage disequilibrium among polymorphisms rs4818, rs4680 and rs4633.**

| **Markers** | **D'** | **LOD** | **$r^2$** |
|---|---|---|---|
| **rs4818 – rs4680** | 0.798 | 9.23 | 0.637 |
| **rs4818 – rs4633** | 0.791 | 8.61 | 0.603 |
| **rs4680 – rs4633** | 1.0 | 19.49 | 0.964 |

**Table S2-4. Genotype frequencies of rs4818 and rs4680 in healthy controls (HC) and patients with CFS/FM.**

| Marker | Frequency | Frequency patients | Frequency healthy controls | Chi Square | P-value |
|---|---|---|---|---|---|
| **rs4633** | | | | | |
| TT | 14.8% | 14.2% | 15.4% | 2.529 | 0.287 |
| CT | 66.7 | 75.0% | 57.7% | | |
| CC | 18.5% | 10.7% | 26.9% | | |
| **rs4818** | | | | | |
| CC | 20.8% | 18.5% | 19.2% | 0.072 | 0.965 |
| GC | 56.6% | 55.6% | 57.7% | | |
| GG | 22.6% | 22.2% | 23.1% | | |
| **rs4680** | | | | | |
| AA | 14.8% | 14.3% | 15.4% | 2.930 | 0.403 |
| GA | 68.5% | 75.0% | 61.5% | | |
| GG | 18.5% | 10.7% | 23.1% | | |

**Table S2-5. Haplotype association in healthy controls (HC) and patients with CFS/FM.** Chi Square tests were performed via SPSS to evaluate differences in haplotype frequency distribution between healthy controls and patients with CFS/FM as a whole instead of comparing the frequency of a single haplotype separately (as was done by Haploview). Enzyme activity is based on previous research. [1]

| Haplotype | | | COMT Enzyme activity | Frequency | Frequency CFS/FM | Frequency HCs | Chi-Square | P-value |
|---|---|---|---|---|---|---|---|---|
| **rs4633** | **rs4818** | **rs4680** | | | | | | |
| **C** | **G** | **G** | High | 45.9% | 42.4% | 49.7% | 0.582 | 0.445 |
| **T** | **C** | **A** | High-intermediate | 43.1% | 44.1% | 42.0% | 0.051 | 0.821 |
| **T** | **G** | **A** | Intermediate | 5.0% | 7.6% | 2.2% | 1.646 | 0.199 |
| **C** | **C** | **G** | Intermediate-low | 5.0% | 5.9% | 4.2% | 0.161 | 0.688 |
| | | | | | | **Overall distribution** | 3.874 | 0.194 |

*Validation of inflammatory factors*

Validation of cytokines was performed is serum, using ELISA human assay kit (Thermofisher, Invitrogen Inc., USA), following manufacturer's protocol. A standard curve for each cytokine was designed by diluting the standards provided in the kit, which contain known concentration of each cytokine. Two blank wells per plate were included and the average signal of those used to correct standard and sample concentrations.

- IL-6 was detectable in 99/108 samples. We used Invitrogen Human IL-6 High Sensitivity ELISA kit. According to the kit's User Guide, sensitivity was determined to be 0.03 pg/mL, intra-assay reproducibility is 4.9%, and inter-assay reproducibility is 6%. Standard curve: $r^2$= 0.9951.
- IFN-gamma was detectable in 94/108. We used Invitrogen Human IFN-y ELISA kit. According to the kit's User Guide, sensitivity was determined to be 0.99 pg/mL, intra-assay reproducibility is 4.5%, and inter-assay reproducibility is 5.7%. Standard curve: $r^2$= 0.9774.
- TGF-beta was detectable in 102/108. We used Invitrogen Human TGF-B1 ELISA kit. According to the kit's User Guide, sensitivity was determined to be 8.6 pg/mL, intra-assay reproducibility is 3.2%, and inter-assay reproducibility is 4.9%. Standard curve $r^2$= 0.9578.

**Bibliography for Supplementary Material S2**


[1] Nackley AG, Shabalina SA, Tchivileva IE, Satterfield K, Korchynskyi O, Makarov SS, Maixner W, Diatchenko L. Human catechol-O-methyltransferase haplotypes modulate protein expression by altering mRNA secondary structure. Science 2006;314:1930–1933.

[2] Swift-Scanlan T, Smith CT, Bardowell SA, Boettiger CA. Comprehensive interrogation of CpG island methylation in the gene encoding COMT, a key estrogen and catecholamine regulator. BMC Med Genomics 2014;7:5. doi:10.1186/1755-8794-7-5.

[3] Tenhunen J, Salminen M, Lundström K, Kiviluoto T, Savolainen R, ulmanen I. Genomic organization of the human catechol O-methyltransferase gene and its expression from two distinct promoters. Eur J Biochem 1994.

## SUPPLEMENTARY MATERIAL (S3)
## STABILITY AND VARIABILITY OF BIOLOGICAL MEASURES

**Table S3. Interclass Correlation Coefficient (ICC) to test between-assessment stability and variability of biological measures – DNA methylation and cytokine expression.** $\alpha$: Cronbach's Alpha. #: statistical significance for ICCs.

| | ICCs | |
|---|---|---|
| | **$\alpha$** | **Sig.** |
| **DNA methylation (%)** | | |
| MB-COMTa | .465 | .015# |
| MB-COMTb | .141 | .153 |
| MB-COMTc | .395 | .035# |
| Mean MB-COMT | .462 | .013# |
| S-COMTa | .708 | .000# |
| S-COMTb | .657 | .000# |
| S-COMTc | .070 | .396 |
| Mean S-COMT | .673 | .000# |
| Exon IV | .187 | .227 |
| **Cytokine expression** | | |
| IL-6 (pg/ml) | .620 | .001# |
| IFN-$\gamma$ (pg/ml) | .583 | 004# |
| TGF-$\beta$ (ng/ml) | .643 | 000# |
