# Supplementary material for: Genetic and epigenetic regulation of Catechol-O-methyltransferase in relation to inflammation in chronic fatigue syndrome and Fibromyalgia
Source: J Transl Med. 2022 Oct 25;20:487. doi: 10.1186/s12967-022-03662-7 (PMC9598022; doi:10.1186/s12967-022-03662-7)
Supplement: Supplementary file 2 — Supplementary Material 2 [file 12967_2022_3662_MOESM2_ESM.docx]

To Prof. Francesco M. Marincola,

Editor of the *Journal of Translational Medicine,*

**Subject: submission or revised manuscript (JTRM-D-22-03438) – Genetic and epigenetic regulation of Catechol-O-methyltransferase in relation to inflammation in Chronic Fatigue Syndrome and Fibromyalgia**

Dear Prof. Francesco M. Marincola,

Thank you for considering our manuscript for publication and for giving us the opportunity to improve the article, implementing reviewers’ comments and suggestions. Please find below the reviewer reports, with point-to-point responses, as requested.

Do not hesitate to contact us if additional information is needed;

Thanks for your consideration,

Kind regards,

On behalf of all authors,

**Andrea Polli**

Department of Physiotherapy, Human Physiology and Anatomy

Pain in Motion Research group - Vrije Universiteit Brussel (VUB)

Department of Public Health and Primary Care, Katholieke Universiteit Leuven (KUL)

Email: [andrea.polli@kuleuven.be](mailto:andrea.polli@kuleuven.be)

**REVIEWER REPORTS**

**REVIEWER 1**

**Reviewer #1: *Dear Author,***

***Your manuscript is interesting to read however i have queries particulary in relation to methodology.***

Dear Reviewer,

thanks for taking the time and read our manuscript. Your valuable comments will definitely help improving it.

***Q/ In subsection 'participants' of methodology, kindly explain the line no 45 to line no.48 in detail (Healthy controls were recruited). The study seems biased for subject recruitment inclusing only female population. The higher prevalence of CFS/FM in females can't not be considered as strong ground to exclude male population.***

Thanks for your comment. The prevalence of CFS and FM is higher in women (70-80%). In addition, important differences exist between sexes, with regards to immune functions and mechanisms. As our study was related to immune functions, we preferred to include only women. If we included men too, we had to drastically increase the sample size, which we were not in a position to do. We added a sentence in the discussion session, in order to highlight this limitation. It now reads as follows: “*One other limitation concerns the generalizability of our results. As explained, given prevalence of CFS and FM is much higher in women (70-80%)^35^ and because important sex differences exist for pain and immune function,^36^ we included only women in the present study. This might mean that our finding might differ in men with CFS/FM, and we should be careful when trying to generalize current results to a male population with CFS/FM.”*

***Q/ the sample size calculation needs more explaination. in its current form its vague.***

Thanks for your comment. Due to word limitation, we tried to keep the text as short as possible, but we agree some additional explanation is needed. We expanded the paragraph, which now reads as follows: *“As no previous study explored COMT DNA methylation in people with CFS/FM and a repeated measure design, we used one study by Kyoung-Sae et al.^25^ to calculate the effect size. Authors explored DNA methylation of COMT in healthy controls and people with clinical depression using our same methodology (PCR-pyrosequencing in the promoter region of MB-COMT). They found a significant between-group mean difference of 1.36% +/- 0.9 (F-test: 28.024, partial n^2^ :0.167). Based on that information, G*Power 3.1 calculated an effect size of f=0.45. We then calculated the sample size needed to detect an effect size of f=0.45 at the significance level of α=5% and a power of 80%, using a repeated-measure design with 2 groups and 2 measurements.^38^ The total sample size needed was 52 participants. This is also in line with a number of previously published studies measuring variability and clinical relevance of DNA methylation using a candidate gene approach. As summarised by Jones et al. ^13^, most studies show that a mean difference of 1-2% in DNA methylation is sufficient for inducing a significant downstream effect on gene expression.^13^”*

***Q/ What was the intra-obsever bias in questainnaire validation?.***

*Thanks for this comment, psychometric properties of the questionnaires, including intra- and inter-rater reliability were excellent, as reported by the two studies we cited.^29,43^We added this information into the manuscripts.*

***Q/ Too smany subtitles in result part compromises the logical flow. It is highly recommended to omitt excessive subtitles.***

Thanks for the comment. We merged two paragraphs and now the result section includes only 3 subsections. We believe it reads better now and it is easier to follow the logical flow.

***Q/ The manuscript need to be edited by native Englsih speaker for English language and grammar.***

Thanks for your comment. The manuscript has been completely reviewed by a native English speaker.

**REVIEWER 2**

***Reviewer #2: The manuscript was well designed.***

***Also, the paper was nicely written and the authors reviewed suitable references to corroborate their hypothesis. There some concerns needs to be improved.***

Dear Reviewer,

thanks for taking the time and read our manuscript, and for your positive and valuable comments.

***Q/ 1. It is suggested to add new research studies about the toxicological concerns.***

Thank you for this very interesting comment. At this stage, we believe we are not in a position to propose any pharmacological intervention related to COMT mechanisms, nor to speculate on toxicological concerns. More research is needed to arrive to such stage. However, higher COMT methylation would suggest reduced degradation of catecholamines and thus higher expression and availability of adrenaline, noradrenaline, and dopamine. Higher expression of catecholamines would potentially have broad downstream effects on several mechanisms, including oxidative stress and mitochondrial functioning. As you suggested in your comments, we are expanding our discussion session to include your suggestions. Please refer to the following answers.

***Q/ 2.what is the suggestion of this study for future works?***

Dear reviewer, thanks for this suggestion. We targeted implemented your comment in the discussion section, where we added a new paragraph that reads: *“High levels of catecholamine expression can also have broad effects on other biological mechanisms, including oxidative stress and mitochondrial function. Mitochondrial dysfunctions and metabolic alterations have been repeatedly observed in people with CFS/FM. Research is not yet conclusive, and the cause of such metabolic alterations is unclear. Our results might suggest that mechanisms related to the autonomic nervous system might play a role. Further research should focus on the relation between catecholamine expression and mitochondrial function to provide potentially crucial pathophysiological mechanisms. This might be especially interesting in light of recent advancements in the field of biomaterials and nano-antioxidants, which appear to interact with inflammatory genes.”*

***Q/ 3.Please discuss about the using of new biomaterials with new technologies including nanoantioxidants.***

Dear reviewer, we added a few sentences introducing the potential relevance of nano-antioxidants. It reads as follows: *“Further research should focus on the relation between catecholamine expression and mitochondrial function to provide potentially crucial pathophysiological mechanisms. This might be especially interesting in light of recent advancements in the field of biomaterials and nano-antioxidants, which appear to interact with inflammatory genes.”*

***Q/ 4.It will be useful if authors discuss the possible role of mitochondria targeting in this work.***

Dear reviewer, thanks for this suggestion. We targeted implemented your comment in the discussion section, where we added a new paragraph that reads: *“High levels of catecholamine expression can also have broad effects on other biological mechanisms, including oxidative stress and mitochondrial function. Mitochondrial dysfunctions and metabolic alterations have been repeatedly observed in people with CFS/FM. Research is not yet conclusive, and the cause of such metabolic alterations is unclear. Our results might suggest that mechanisms related to the autonomic nervous system might play a role. Further research should focus on the relation between catecholamine expression and mitochondrial function to provide potentially crucial pathophysiological mechanisms. This might be especially interesting in light of recent advancements in the field of biomaterials and nano-antioxidants, which appear to interact with inflammatory genes.”*

***Q/ 5.Please add these references for your discussion part of manuscript and bold your study novelty:***

***-DOI: 10.1186/2008-2231-21-28***

***-DOI: 10.1371/journal.pone.0250547***

Dear reviewer, thanks for this comment. We have added the two references in the following two paragraphs:

*1. “Further research should focus on the relation between catecholamine expression and mitochondrial function to provide potentially crucial pathophysiological mechanisms. This might be especially interesting in light of recent advancements in the field of biomaterials and nano-antioxidants, which appear to interact with inflammatory genes.”*

*2. “Finally, we feel the urge to highlight that translational research is extremely complex in nature. Dealing with often remarkably variable biological markers is challenging. This is especially true when researchers attempt to better understand complex and heterogeneous conditions such as CFS/FM, where multiple complex mechanisms are likely at play and influence each other. We recommend future research to employ a repeated-measure design to control for biomarkers variability and within-subject changes. In addition, a better characterization of the subjects enrolled in the research is warranted. Cognitive, psychological, and social aspects can all interact with biological mechanisms and needs to be considered when designing a clinical study.”*

**REVIEWER 3**

***Reviewer #3: The authors present a manuscript regarding Genetic and epigenetic regulation of Catechol-O-methyltransferase in relation to inflammation in Chronic Fatigue Syndrome and Fibromyalgia***

***The authors indicate COMT regulates nociceptive processing and inflammation, resulting in Chronic Fatigue Syndrome and Fibromyalgia.  And that, fifty-four participants (28 patients with CFS/FM and 26 controls) were assessed twice within 4 days that included clinical questionnaires and neurophysiological assessments.  The authors found MB-COMT DNA methylation is an independent factor contributing to the pathophysiology of CFS/FM.***

Dear reviewer, thanks for your time reviewing our manuscript. We implemented your comments and are confident that it now has improved.

***Q/ There are come concerns with the manuscript methodology/***

***"After the first and tenth pulse, the participant had to rate her pain on the visual analog scale" . Were not all genders included?***

Dear reviewer, thanks for your comment. The prevalence of CFS and FM is higher in women (70-80%). In addition, important differences exist between sexes, with regards to immune functions and mechanisms. As our study was related to immune functions, we preferred to include only women. If we included men too, we had to drastically increase the sample size, which we were not in a position to do. We added a sentence in the discussion session, in order to highlight this limitation. It now reads as follows: “*One other limitation concerns the generalizability of our results. As explained, given prevalence of CFS and FM is much higher in women (70-80%)^35^ and because important sex differences exist for pain and immune function,^36^ we included only women in the present study. This might mean that our finding might differ in men with CFS/FM, and we should be careful when trying to generalize current results to a male population with CFS/FM.”*

***Q/ In the data series (Figure 1 and Figure 2), there is very little to no significant reporting of % DNA methylation, in any group.***

Thanks for pointing this out. Given the high variability of biomarkers and the possibility of occurring in false positive findings, we decided to employ conservative statistical models (e.g. Bonferroni correction, p-value set at 0.008, etc.). This necessarily gives less statistically significant findings, but we do believe the significant findings we report are likely to be of clinical relevance. All statistically significant results are highlighted in table 1, 2, and 3.

***Q/ In certain parameters there is an apparent misunderstanding of seam biology.  The covariates measured in the CSL total list , in fact, show greater values in CFS.FM as compared to HC group. However, CFS has less IFN-y levels,***

Dear reviewer, thanks for your interesting comment. We were indeed surprised to find such result ourselves. As you correctly point out, we were expecting IFN-y to be higher in CFS. However, we found a lower expression of IFN-y. There is not much we can do about it but report the result we found. We also tried to discuss this finding in the discussion session, and found that IFN-y, at low doses, can actually cover an anti-inflammatory effect (as reported in previous studies in CFS too), which might be the case in our sample. The paragraph reads as follows: *“Another significant result was a lower expression of IFN-γ in patients. IFN-γ has long been known as an inflammatory cytokine, e.g. by inducing the production of other cytokines such as IL-6 and TNF-α.^30^ However, other observations highlight the role of IFN-γ as master regulator of immunity.^48^ Low levels of IFN-γ (below 50 pg/ml) exert a potent anti-inflammatory effect.^8^ Our results seems to confirm the anti-inflammatory role of IFN-γ, as already showed by previous research in CFS.^2^ ”*

***Q/ The indication is that methylation of the COMT promoter is more associative to CFS than IFN-y, however the authors have not performed a full cytokine profile of their data set.***

Thanks for this comment. We unfortunately were not in a position to perform a full cytokine profile. Therefore, we selected some of the cytokines that had been previously associated with CFS/FM. However, we acknowledge that full cytokine profiling would be preferable when possible, and stated this in the discussion session, which now reads: *“Our results seems to confirm the anti-inflammatory role of IFN-γ, as already showed by previous research in CFS.^2^ However, given some conflicting results present in literature, further research is needed to confirm our findings, better if employing, when possible, assays for full cytokine profiling.”*

***Q/ In the data series only three polymorphisms are shown, so it is not clear as to the relevance of this data, and why more variants are not included.***

Dear reviewer, we could not explore all possible SNPs in the COMT gene. Therefore, we selected the three that had been previously shown to be clinically relevant. The three SNPs we selected are in fact passed in haplotypes and dramatically influence COMT enzyme expression and activity. As reported in the introduction: *“Three polymorphisms – rs4633, rs4818, and rs4680 – cover particular importance and influence S-COMT enzyme activity.^33,41^ These polymorphisms are inherited together in different haplotypes, which in turn influence COMT activity and clinical presentations.^6^”*

***Q/ The reviewer disagrees that CPM is significantly different in CFS vs HC.  (Table 2).  The authors should report another behavior of CFS effects besides CPM***

Dear reviewer, thanks for your comments. We do not disagree that CPM is significantly different. Our results do not simply support such statement and we have to consider this. We in fact included also a full range of clinical symptoms in our study (CSL questionnaire). In addition, we added a paragraph in the discussion to highlight the importance of a full clinical and behavioural assessment when designing translational research. It reads as follows: *“Finally, we feel the urge to highlight that translational research is extremely complex in nature. Dealing with often remarkably variable biological markers is challenging. This is especially true when researchers attempt to better understand complex and heterogeneous conditions such as CFS/FM, where multiple complex mechanisms are likely at play and influence each other. We recommend future research to employ a repeated-measure design to control for biomarkers variability and within-subject changes. In addition, a better characterization of the subjects enrolled in the research is warranted. Cognitive, psychological, and social aspects can all interact with biological, behavioural, and psychophysical assessments and needs to be considered when designing a clinical study.”*
